# The Spread of SARS-CoV-2 Infection Among the Medical Oncology Staff of ASST Spedali Civili of Brescia: Efficacy of Preventive Measures

**DOI:** 10.3389/fonc.2020.01574

**Published:** 2020-08-18

**Authors:** Alberto Dalla Volta, Francesca Valcamonico, Rebecca Pedersini, Carla Fornaro, Valeria Tovazzi, Sara Monteverdi, Alice Baggi, Francesca Consoli, Vittorio Domenico Ferrari, Salvatore Grisanti, Elisabetta Conti, Vito Amoroso, Paolo Bossi, Alfredo Berruti

**Affiliations:** Medical Oncology Unit, Department of Medical and Surgical Specialties, Radiological Sciences, and Public Health, University of Brescia at ASST Spedali Civili, Brescia, Italy

**Keywords:** SARS-CoV-2, COVID-19, oncology, prevention, Lombardy, Italy, healthcare

## Abstract

Patients with cancer are at a higher risk of developing serious disease-related complications in case of contracting SARS-CoV-2. Oncology units should implement all possible preventive measures to reduce the risk of viral transmission by healthcare professionals (HCPs) to patients. We conducted a surveillance for SARS-CoV-2 infection among the staff members of the Medical Oncology Unit of ASST Spedali Civili in Brescia, one of the Italian areas most affected by the SARS-CoV-2 pandemic. The aim of this study was to demonstrate whether the recommended preventive measures, promptly implemented by the unit, have been effective in reducing the spread of the virus among the HCPs. Between February 24 and May 19, 2020, SARS-CoV-2 infection was detected in 10 out of 76 healthy HCPs (13%). Six of them developed a symptomatic disease, leading to home quarantine, and four remained asymptomatic. The infection was revealed when a serology test was performed on all staff members of the unit. In seven HCPs, in which it was possible to trace the person-to-person infection, the contagion occurred as a result of unprotected contacts or partially protected with surgical masks. In particular, four asymptomatic HCPs did not stop working, but a widespread outbreak in the unit was avoided. Adherence to the recommended preventive strategies, in particular, wearing of surgical masks by both the HCPs and the patients, is effective in reducing and preventing the viral spread.

## Introduction

Coronavirus SARS-CoV-2 infection and coronavirus disease (COVID-19) emerged in China between October and December 2019 (1), and in the subsequent months, it spread rapidly throughout the world. The World Health Organization declared the outbreak as a public health emergency of international concern on 30 January 2020 and as a pandemic on 11 March 2020. Italy was the first European nation to be hardly affected by the epidemic. The first infectious cluster in Italy was identified in Lombardy on February 21, 2020, and since then Lombardy was the Italian region that recorded the highest number of people infected with the virus. The province of Brescia (1.264 million population) has been one of the most affected areas in Lombardy. As of May 19, 2020, the province had 14,199 confirmed cases and 2,432 deaths (2). To manage the coronavirus emergency, the ASST Spedali Civili of Brescia, one of the largest hospitals in Italy, has reserved more than 800 beds to patients suffering from serious complications of the disease.

Since last February 24, 2020, the Medical Oncology Unit of ASST Spedali Civili in Brescia put in place most of the protective measures that were subsequently acknowledged to be effective in preventing viral infection among healthcare professionals (HCPs) and patients (3–5).

We report in this paper the results of a prevalence survey to assess the extent of transmission of SARS-CoV-2 infection among healthy workers of our oncology unit in the observational period between February 24 and May 19, 2020.

## Subjects and Methods

The staff of the Medical Oncology Unit of ASST Spedali Civili of Brescia is composed of 76 healthy workers: 17 medical oncologists, 12 resident doctors, 37 nurses, four administrative clerks, five data managers, and one psychologist. Nineteen are male and 57 are female; the median age is 46, and none of them suffers from major comorbidities (Table 1). The ward consists of an inpatient unit with 18 beds and a day hospital unit with 22 therapy stations and 10 ambulatory centers. The number of outpatients managed for daily therapy in the pre-pandemic era was 110–120 per day.

**TABLE 1:** Healthcare professionals (HCPs) characteristics.

Gender	M 19 (25%)	F 57 (75%)
Median age	46 (23–69)	
Nurses	37 (48.7%)	
Oncologists	17 (22.4%)	
Residents	12 (15.8%)	
Datamanagers	5 (6.6%)	
Administratives	4 (5.3%)	
Psychologist	1 (1.2%)	
Any symptoms	15 (19.7%)	
Fever	10 (13,1%)	
Cough	5 (6.5%)	
Anosmia/Dysgeusia	5 (6.5%)	
Asthenia	5 (6.5%)	
Asymptomatic	61 (80.3%)	
**SARS-CoV-2 positive**	**10 (13%)**	
Fever	6 (60%)	
Anosmia/Dysgeusia	5 (50%)	
Cough	4 (40%)	
Headache	4 (40%)	
Asthenia	3 (30%)	

From February 24, 2020, onward, several mandatory measures were adopted to prevent, as much as possible, SARS-CoV-2-related nosocomial cross-infections between patients and staff members. One important tool we looked at was the application of telemedicine.

Patients receiving oral therapies were managed at home through telephone contacts or video calls. The necessary blood tests were performed in outpatient clinics outside the hospital, and the results were sent by fax or email. The drugs were delivered outside the ward to patients or family members. Follow-up visits were accordingly converted to phone calls, and medical reports were sent to the patients *via* email.

Access to oncologic structures was limited and/or denied for visitors and caregivers, either for outpatient visits or day hospital and ward admissions.

With these measures, the number of admitted patients to our day hospital decreased to 50–70 every day and was carefully distributed over the day in order for them to avoid staying long in the waiting room and to maintain safety distances among patients.

For the admitted patients needing intravenous therapies, essential personal protective measures, such as a surgical mask and disinfectants, were provided. A triage performed by a trained nurse was introduced, which consisted of questions regarding the presence of fever, cough, dyspnea, unexplained fatigue, anosmia, dysgeusia, headache, nasal congestion, conjunctival congestion, sore throat, diarrhea, nausea, and vomiting and of measurement of pulse oximetry and body temperature. Patients with suspected signs and symptoms of SARS-CoV-2 infection were immediately advised to leave the day hospital for appropriate investigations to be performed at dedicated units.

All HCPs had to wear the following personal protective equipment: surgical mask and powder-free, non-sterile nitrile gloves—and all of them washed their hands frequently for at least 20 s. Nurses devoted to therapy administration also wore disposable medical gowns. Face shields and filtering facepiece (FFP) 2 masks were adopted in the management of patients with symptoms and signs of SARS-CoV-2 infection.

Regarding the work activities, multidisciplinary board conferences have been converted to telematic meetings, and counseling of patients in other hospital wards has been managed by phone, whenever feasible.

No physician or nurse was involved on duties in SARS-CoV-2 dedicated wards in order to avoid risk of infection for both patients and colleagues.

As per hospital instructions, a nasopharyngeal swab was performed on all health workers with suspected symptoms related to SARS-CoV-2 infection. Those with known infection remained at home on quarantine or were referred to dedicated units if necessary. All the others remained at work.

On April 27, 2020, all healthcare providers underwent a serological test for the presence of IgG antibodies directed against viral spike S1-2 glycoproteins (DiaSorin; normal <12 AU/ml, uncertain 12.0–15.0 AU/ml, and positive >15 AU/ml).

## Results

**FIGURE 1:**
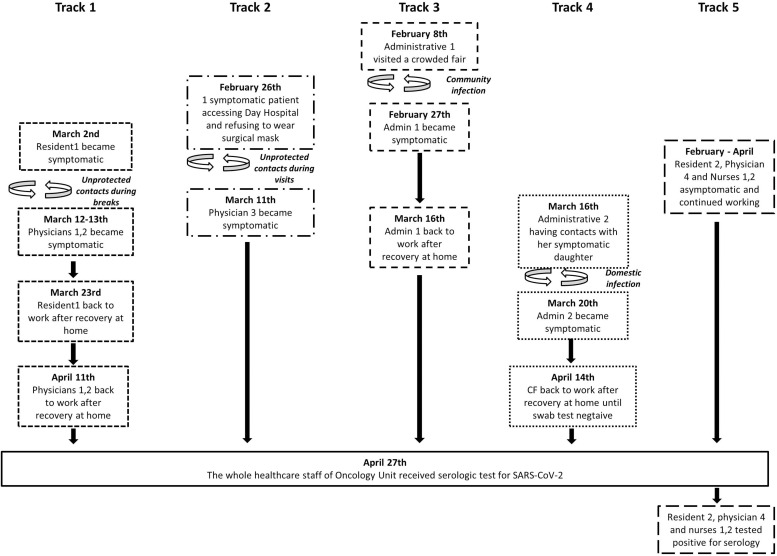
Flow chart of likely infection spread among the healthcare professionals (HCPs) of the Oncology Unit.

In the observation period, six HCPs (8%; four physicians and two administrative clerks), developed a clinically evident SARS-CoV-2-associated disease. The symptoms and signs consisted of fever (83%), headache (67%), cough (67%), and asthenia (50%) (Table 1). All of them remained at home on quarantine for a median of 24.5 days (range 14–50), and none needed hospitalization for severe disease complications. In these workers, we retrospectively evaluated specific person-to-person transmission events. One resident reported symptoms typical for SARS-CoV-2 infection on March 2, 2020 and remained at home until 4 days after the complete resolution of symptoms. She came back to work on March 23, 2020, and her nasopharyngeal swab result was negative. Before the typical symptoms became evident, she was long in contact with two medical oncologists during coffee and lunch breaks, which were occasions when the surgical mask was removed. These two physicians developed a symptomatic disease on March 12 and 13, 2020, leading to home quarantine until they recovered from the symptoms and their nasopharyngeal swab result came out negative (Figure 1). The two administrative clerks developed COVID-19 on February 27 and March 20, 2020 and stayed at home for quarantine until completely recovered and had a negative result of their swab. One of them was most likely infected during a visit to a crowded fair on February 8, 2020, while the second developed a symptomatic SARS-CoV-2 infection after having been in contact with her daughter who became sick on March 14, 2020 (Figure 1). The last physician was likely infected on February 26, 2020 during a visit of a symptomatic patient who refused to wear a surgical mask.

As a result of the serological test on April 27, 2020, additional four health workers, one oncologist, one resident doctor, and two nurses tested positive for IgG antibodies. Three of them were always asymptomatic and one suffered with mild flu-like symptoms. We could retrospectively assess the possible person-to-person transmission for one of these healthcare professionals who had performed repeated visits between February 5 and 20, 2020 to a patient who complained with mild aspecific symptoms and further developed a full-blown symptomatology of the SARS-CoV-2 disease. These visits were made without adequate protection since they occurred in a period prior to the first case officially described in Lombardy. Today we know that the virus circulated in the Lombardy population weeks before the official discovery. Conversely, contact could not be tracked back as regard to the other physician and the two nurses. Of note is that the median serum level of specific IgG was 31.5 AU/ml (range 15–67.2) for the symptomatic HCPs and 18.4 AU/ml (range 15–27.6) for the asymptomatic HCPs, although the small sample size does not allow any conclusion regarding the correlation between the IgG levels and the clinical presentation of COVID-19.

Overall, 10 HCPs out of 76 (13%) have been certainly infected, of which six developed the disease and four remained asymptomatic. As outlined in Figure 1, in the seven HCPs in which it was possible to trace the cause of the infection, the contagion occurred as a result of unprotected contacts or partially protected by personal protective equipment. Figure 1 clearly depicts that the SARS-CoV-2 infection was confined to individuals and in one small group of HCPs.

## Discussion

During the SARS-CoV-2 pandemic, oncology units should adopt all possible precautions to reduce the spread of the virus among patients and HCPs (4). The adoption of telemedicine, which was probably underused before the current emergency, helped us to avoid overcrowding in the Oncology Unit, without causing any reduction in the quality of care, in line with a recent review of the literature (6). Physical distancing of 1 m or more and the use of face masks have been shown, in a recent meta-analysis of data from heterogeneous healthcare and community settings (7), to significantly protect against the risk of transmission of betacoronaviruses. Several papers have been published in recent weeks, suggesting the protective measures to be taken by the oncology staff (3, 4, 8, 9), but no data on their efficacy in this specific setting is available so far. The Medical Oncology Unit of ASST Spedali Civili of Brescia is located in one of the Italian areas that were most affected by the SARS-CoV-2 infection, and the hospital had to allocate the majority of its beds to patients with severe virus-related diseases. One problem that we had at the onset of the pandemic was whether the surgical mask was sufficient to protect the staff and the patients from the spread of the virus or the FFP2 masks with a deeper filtration efficacy would have been more effective (10). As these latter devices were not available in sufficient quantity, only surgical masks, together with general protective measures, were routinely adopted by patients and staff. Moreover, due to the high level of SARS-CoV-2 shedding in the upper respiratory tract, asymptomatic transmission is the Achilles’ heel of current strategies to control the spread of the infection (11). Although SARS-CoV-2 infection was later found on four asymptomatic professionals who did not stop working, a widespread outbreak, as occurred in a skilled nursing facility (12), was avoided in our medical oncology unit. This is possibly due to the protection given by the adoption of the use of personal protective equipment, even if the possibility that asymptomatic carriers could have a reduced viral load in comparison with symptomatic HCPs cannot be ruled out.

Our experience demonstrates that adherence to the recommended preventive strategies, in particular, wearing of surgical masks by both the health workers and the patients, is effective in reducing and preventing the spread of the virus. By contrast, the protection derived from wearing a surgical mask is probably not effective enough if this is worn by the HCPs but not by the patients. The more efficient FFP2 masks, which are less well-tolerated (13, 14), could not be a mandatory device in routine patient management within an oncology unit.

## Data Availability Statement

The raw data supporting the conclusions of this article will be made available by the authors, without undue reservation.

## Ethics Statement

The studies involving human participants were reviewed and approved by Comitato Etico di Brescia – ASST Spedali Civili di Brescia. The patients/participants provided their written informed consent to participate in this study.

## Author Contributions

AD and FV: conceptualization, data curation, investigation, formal analysis, writing-original draft, writing-review, and editing. RP, VF, SG, VA, and PB: data curation, writing-review, and editing. CF, ABa, FC, and EC: data curation. VT and SM: writing-review and editing. ABe: conceptualization, formal analysis, methodology, project administration, supervision, writing-original draft, writing-review, and editing. All authors contributed to the article and approved the submitted version.

## Conflict of Interest

The authors declare that the research was conducted in the absence of any commercial or financial relationships that could be construed as a potential conflict of interest.
